# Applications of the PMO platform to genetic diseases

**DOI:** 10.1186/1750-1172-10-S2-O34

**Published:** 2015-11-11

**Authors:** Ryszard Kole

**Affiliations:** 1 Sarepta Therapeutics, Cambridge, MA 02142, USA

## 

Genetic diseases are caused by a variety of mutations some of which lead to aberrant splicing of pre- mRNA and prevent proper mRNA translation of essential proteins or lead to translation of undesirable proteins. Such mRNA defects can be frequently repaired by appropriate targeting of oligonucleotides to modify splicing pathways and restore correct translation of desirable proteins.

Hutchinson-Gilford progeria syndrome (HGPS), the main topic of this conference, other laminopathies, as well as diseases such as Duchenne muscular dystrophy (DMD) and thalassemia, are amenable to splicing manipulation or exon skipping. It has been shown in cell culture, in animal disease models or in case of DMD in clinical trials that oligonucleotides targeted to appropriate pre- mRNA splicing elements can restore correct splicing and allow production of desirable proteins, i.e. dystrophin in DMD, beta-globin in thalassemia, or reduce the level of harmful proteins, such as progerin in HGPS.

Sarepta Therapeutics develops phosphorodiamidate morpholino oligomers (PMOs) and their derivatives as potential drugs for the treatment of rare diseases. After more than three years of treatment with PMO drug candidate, eteplirsen, stability of respiratory functions was observed and the results of the 6-minute walk test (6MWT) at 168 weeks showed continued ambulation across all patients evaluable on the test. Some decline in distance walked was observed since the week 144 time point. No significant treatment related adverse events were observed over the three-year course of this study.

